# Left atrial blood stasis by 4D flow MRI correlates with stroke risk estimation by CHA2DS2-VASc score

**DOI:** 10.1186/1532-429X-16-S1-O51

**Published:** 2014-01-16

**Authors:** Daniel C Lee, Michael Markl, Jacob Fluckiger, Jason Ng, James C Carr, Jeremy D Collins, Jeffrey J Goldberger

**Affiliations:** 1 Northwestern University Feinberg School of Medicine, Chicago, Illinois, USA; 2 GE Healthcare, Waukesha, Wisconsin, USA

## Background

Stroke is the most serious complication of atrial fibrillation (AF). Of the estimated 600,000 ischemic strokes that occur each year in the United States, 15-20 percent are thought to be secondary to AF. Blood stasis is a key factor responsible for thrombus formation in the left atrium (LA). Studies utilizing transesophageal echocardiography have shown that peak left atrial appendage flow velocity < 0.2 m/s is an independent risk factor for stroke in AF. 4D Flow MRI may improve prediction of stroke risk in AF patients by examining 3D flow patterns across the entire LA volume over multiple phases spanning the cardiac cycle. We hypothesized that LA stasis measured by 4D flow MRI correlates with stroke risk estimated by the CHA2DS2-VASc clinical risk score in patients with AF.

## Methods

We performed 4D flow MRI in 31 patients with documented AF, 21 were in sinus rhythm at the time of imaging (AF-sinus) and 10 were still in AF (AF-afib). Each subject underwent standard cardiac protocols on 1.5T and 3T MR systems (Siemens Avanto, Aera, Skyra, Erlangen, Germany) including CINE images to determine cardiac function. In addition, ECG and navigator gated free breathing 4D-Flow MRI was performed for each subject (velocity sensitivity - 100-150 cm/s, spatial resolution = 2.5-3.0 mm in plane, 3.0-3.5 mm slice thickness, temporal resolution = 37.6-41.6 ms). Data analysis included noise filtering as well as correction for Maxwell terms, eddy currents, and velocity aliasing. LA volume was manually segmented to enable analysis of all LA voxel velocities over multiple phases spanning the cardiac cycle. The top two tertiles of LA velocities were used for analysis, although results were similar if 100% of the velocities were used. LA flow for each patient was quantified by mean LA velocity and the percentage of LA velocities > 0.2 m/s (incidence). CHA2DS2-VASc scores were calculated for each patient (1 point each for congestive heart failure, hypertension, age 65-75, diabetes, vascular disease or female gender; 2 points for prior stroke/thromboembolism, or age ≥ 75).

## Results

**Figure 1:**
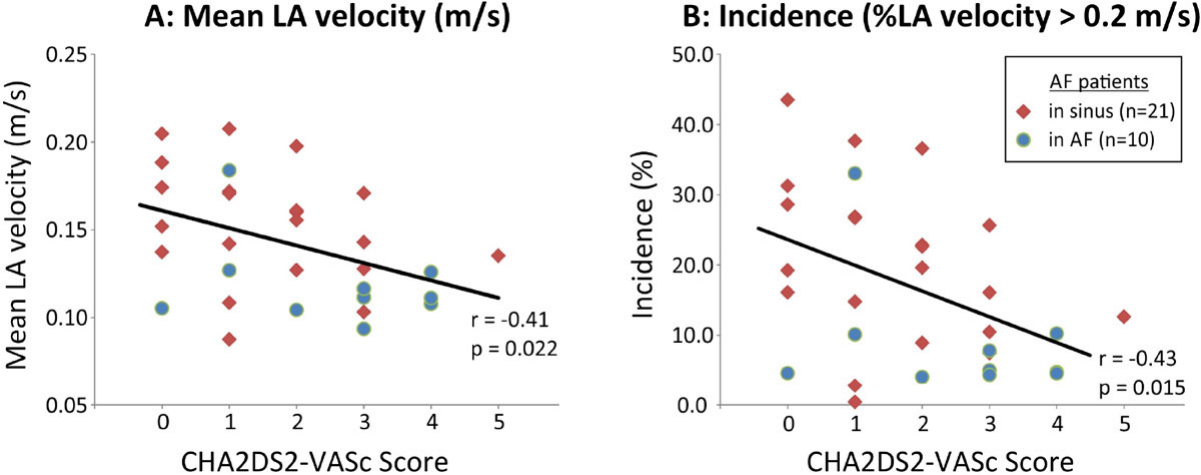
**Left atrial stasis measured by 4D Flow MRI versus CHA2DS2-VASc score**. A: Mean LA velocity (m/s), B: Incidence of LA velocity > 0.2 m/s.

Patient age was 59.7 ± 9.4 in AF-sinus and 68.1 ± 8.6 AF-afib. CHA2DS2-VASc correlated negatively with LA mean velocity (Figure 1A, r = -0.41, p = 0.022) and percentage of LA velocities > 0.2 m/s (Figure 1B, r = -0.43, p = 0.015). The correlation was similar for AF-sinus (r = -0.31) and AF-afib (-0.32).

## Conclusions

Increased LA blood stasis as measured by 4D flow MRI correlates with higher stroke risk as estimated by the CHA2DS2-VASc clinical risk score. Patients with higher CHA2DS2-VASc score have lower LA mean velocity and a lower percentage of LA velocities > 0.2 m/s. That the correlation was modest may be due to limitations in the CHA2DS2-VASc score - which uses epidemiologic risk factors to estimate stroke risk rather than a physiologic parameter such as blood stasis. Further work will help determine whether 4D Flow MRI can more accurately identify AF patients at increased stroke risk, and guide decisions on anticoagulation.

## Funding

This work is supported by funding from the National Institutes of Health (1R21HL113895-01A1 - JJG) and the American Heart Association (12GRNT12080032 - MM).

